# Factors predicting the use of therapeutic hypothermia and survival in unconscious out-of-hospital cardiac arrest patients admitted to the ICU

**DOI:** 10.1186/cc12826

**Published:** 2013-07-23

**Authors:** TW Lindner, J Langørgen, K Sunde, AI Larsen, JT Kvaløy, JK Heltne, T Draegni, E Søreide

**Affiliations:** 1 Department of Anaesthesiology and Intensive Care, Stavanger University Hospital, Norway; 2 Department of Heart Disease, Haukeland University Hospital, Bergen, Norway; 3 Department of Anaesthesiology, Division of Emergencies and Critical Care, Oslo University Hospital, Norway; 4 Department of Cardiology, Division of Internal Medicine, Stavanger University Hospital, Norway; 5 Department of Clinical Medicine, University of Bergen, Norway; 6 Department of Mathematics and Natural Sciences, University of Stavanger, Norway; 7 Department of Anaesthesiology and Intensive Care, Haukeland University Hospital, Bergen, Norway; 8 Department of Research and Development, Division of Emergencies and Critical Care, Oslo University Hospital, Norway

**Keywords:** Out-of-hospital cardiac arrest, therapeutic hypothermia, survival, age, gender, intensive care

## Abstract

**Introduction:**

Therapeutic hypothermia (TH) after out-of-hospital cardiac arrest (OHCA) was adopted early in Norway. Since 2004 the general recommendation has been to cool all unconscious OHCA patients treated in the intensive care unit (ICU), but the decision to cool individual patients was left to the responsible physician. We assessed factors that were associated with use of TH and predicted survival.

**Method:**

We conducted a retrospective observational study of prospectively collected cardiac arrest and ICU registry data from 2004 to 2008 at three university hospitals.

**Results:**

A total of 715 unconscious patients older than 18 years of age, who suffered OHCA of both cardiac and non-cardiac causes, were included. With an overall TH use of 70%, the survival to discharge was 42%, with 90% of the survivors having a favourable cerebral outcome. Known positive prognostic factors such as witnessed arrest, bystander cardio pulmonary resuscitation (CPR), shockable rhythm and cardiac origin were all positive predictors of TH use and survival. On the other side, increasing age predicted a lower utilisation of TH: Odds Ratio (OR), 0.96 (95% CI, 0.94 to 0.97); as well as a lower survival: OR 0.96 (95% CI, 0.94 to 0.97). Female gender was also associated with a lower use of TH: OR 0.65 (95% CI, 0.43 to 0.97); and a poorer survival: OR 0.57 (95% CI, 0.36 to 0.92). After correcting for other prognostic factors, use of TH remained an independent predictor of improved survival with OR 1.91 (95% CI 1.18-3.06; *P *<0.001). Analysing subgroups divided after initial rhythm, these effects remained unchanged for patients with shockable rhythm, but not for patients with non-shockable rhythm where use of TH and female gender lost their predictive value.

**Conclusions:**

Although TH was used in the majority of unconscious OHCA patients admitted to the ICU, actual use varied significantly between subgroups. Increasing age predicted both a decreased utilisation of TH as well as lower survival. Further, in patients with a shockable rhythm female gender predicted both a lower use of TH and poorer survival. Our results indicate an underutilisation of TH in some subgroups. Hence, more research on factors affecting TH use and the associated outcomes in subgroups of post-resuscitation patients is needed.

## Introduction

Based on two landmark publications on mild therapeutic hypothermia (TH) in out-of-hospital cardiac arrest (OHCA) patients [1,2], the International Liaison Committee on Resuscitation (ILCOR) in 2003 issued an advisory statement recommending TH for all unconscious OHCA patients with return-of-spontaneous circulation (ROSC) and an initial shockable rhythm [3]. The committee also suggested cooling unconscious patients with an initial non-shockable rhythm. Hospitals in Norway were early adopters of this new post-resuscitation therapy [4-7]. Since 2004, the Norwegian medical consensus was to provide TH to all unconscious OHCA survivors, regardless of the cause of cardiac arrest, initial rhythm and age, as long as active intensive care unit (ICU) treatment was considered [8]. The first Norwegian experiences with the use of TH were published in 2006 to 2007 [5,7,9]. Although TH was part of a standardised treatment protocol [7], individual therapy was left to physician discretion and bedside judgment. Therefore, treatment in individual patients may not always have followed these national recommendations. On the other hand, the evidence for the use of TH for patients with non-shockable rhythms, OHCA of non-cardiac origin and in older patients has been poor and indirect at best [10]. While TH should mitigate reperfusion injury in the brain, regardless of cause and initial heart rhythm, expanding TH to new patient groups could usurp valuable ICU resources without necessarily improving outcomes.

Based on this background information, we assessed the actual use of TH and the associated outcomes in a health system with early adoption and broad application of TH. More specifically, we analysed which patient and cardiac arrest factors that predicted use of TH and survival to discharge in adult unconscious OHCA patients with both cardiac and non-cardiac aetiologies admitted to the ICU.

## Material and Methods

### Study design and organisation

This is a retrospective observational study of prospectively collected cardiac arrest and ICU registry data from 2004 to 2008 from the Oslo University Hospital Ullevål, Haukeland University Hospital Bergen and Stavanger University Hospital in Norway.

All three centres used a standardised post-resuscitation treatment protocol, including TH for unconscious patients and emergency percutaneous coronary intervention (PCI) for patients with ST-elevation myocardial infarction [7,11]. Prognostication in the study ICUs was primarily done based on clinical examination [4].

Patients were treated in medical, cardiac or general ICUs, and both non-invasive and invasive cooling methods were applied [7,12]. All together, the study sites served a population of approximately 1.3 million people with a combination of TH and emergency PCI.

The Norwegian Emergency Medical Service is regulated by governmental agencies with medical responsibility placed in local health trusts. Emergency dispatch centres coordinate the emergency response of ambulance units, hospital-based and emergency physician-led rapid response units, and general practitioners who are on call in local municipalities [13]. All of these units are called simultaneously when a patient suffers a presumed OHCA [13]. We followed the 2000 and 2005 guidelines from the European Resuscitation Council [14,15], with Norwegian adjustments [14,16].

### Study population

**Figure 1:**
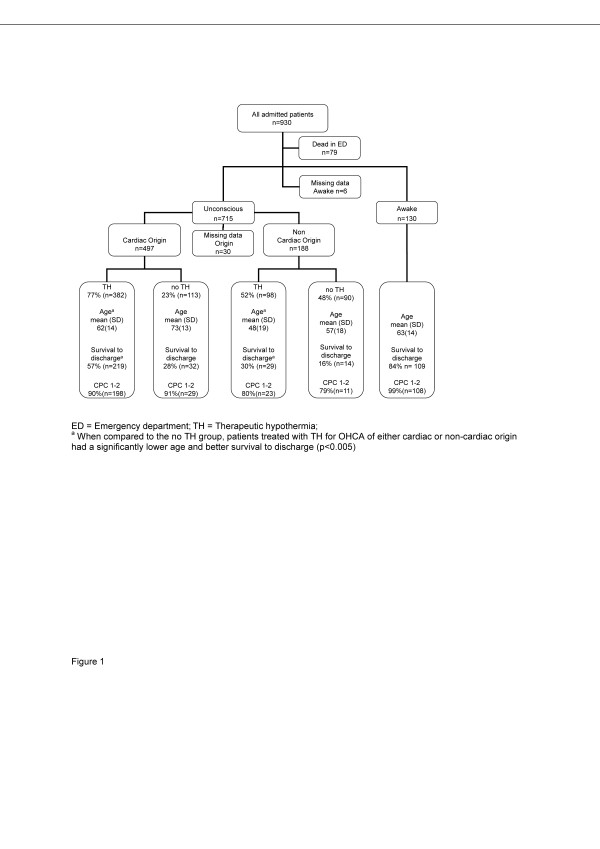
**Flow chart of the 930 patients who had an OHCA and were admitted to the study hospitals between January 2004 and January 2008**.

Between January 2004 and January 2008, we included all adult (age >18 years) OHCA patients admitted with ROSC to an ICU at one of the three study sites (Figure 1). The study population included patients who suffered OHCA from either cardiac or non-cardiac causes. Patients with a trauma-related aetiology were excluded. Only a few patients were admitted to the emergency department with ongoing cardiopulmonary resuscitation (CPR). These patients were included if ROSC was established in the emergency department; otherwise, they were accounted for as prehospital non-survivors and not included (Figure 1). Each of the three hospitals contributed about one-third of the patients included in this study.

### Data collection and definitions

All study sites had a prehospital Utstein-style [17] cardiac arrest registry. Hospital data were obtained from intensive care quality assurance databases and the local Northern Hypothermia Network databases [5,18]. A medical condition was registered as co-morbidity if the patient was under current pharmacological treatment or required medical follow-up due to the disease. The data variables collected in the research database were based on a consensus protocol between the three participating study sites. Each study site was responsible for the verification of the local registered data before they were transferred anonymously to the research database of this study.

TH was registered as performed when a target temperature of 33ºC (± 1ºC) was obtained and kept for 12-24 h. Both external and invasive methods were used to induce and maintain cooling [12,19]. No further details on the use of TH were collected. Good neurological function was defined as a Cerebral Performance Category of 1 or 2 at discharge from the hospital [17]. The calculation of the Cerebral Performance Category was based on information from the hospital charts and follow-up notes.

### Statistical analysis and ethical approval

We used the chi-square test to examine differences in proportions for categorical variables. The nonparametric Kruskal-Wallis test was used to test for differences in means for continuous variables across groups. Logistic regression analysis was used to model the effect of explanatory variables on dichotomous outcome variables. The outcome variables for this study were the use of TH and discharged alive from the hospital. Multivariable logistic regression models were constructed aided by backward stepwise variable selection. We tried both forward and backward selection and the same models were proposed. Variables with a *P *value of <0.25 in univariable analyses were considered as candidates for inclusion in the multivariable model. Goodness of fit for the logistic regression model was verified by the Hosmer-Lemeshow test. Linearity in the log-odds ratio (OR) for continuous explanatory variables was verified by adding smooth spline terms. Possible medical relevant interaction terms between variables were tested.

The following factors were included as explanatory variables in the logistic regression analysis for the use of TH: age, gender, first registered heart rhythm, witnessed OHCA, performed bystander CPR, response time of the emergency medical system, origin of the OHCA, site of the OHCA and study site. The same explanatory variables and the use of TH and co-morbidities (coronary disease, hypertension, heart failure, diabetes mellitus and renal impairment) were included in the logistic regression analysis evaluating survival to hospital discharge.

A propensity score for the probability of a patient to receive TH was calculated with a logistic regression model based on Utstein template variables and medical relevant interaction terms among these. The logarithm of the odds of the propensity score was added as a covariate in the multivariable logistic regression model evaluating survival to discharge from the hospital [20,21].

Missing data occurred in several variables examined in this study. We found it reasonable to assume that missing values are missing at random. This and the fact that the percentage of missing data was low justified using complete case analyses without introducing bias or noticeable reduced power. Information on the missing data is shown in the tables if the missing data comprise >5% of all data. The numbers of complete datasets are reported for the multivariable analyses.

The data were entered into a FilemakerPro7 database (FileMaker, Inc.; USA) and Microsoft^® ^Office Excel. Figures were constructed using SPSS 18.0, Microsoft^® ^Office Excel and Power Point 2003 (Microsoft Corporation, USA). SPSS 18.0 (SPSS, Inc; USA) and R 2.15.0 2 (r-project, USA) were used for statistical analyses. Two-sided *P *values <0.05 were considered statistically significant.

The collection of local data was approved by the Norwegian Social Science Data Service. The Regional Ethics Committee in West Norway (REC West; University of Bergen) stated that use of such data was considered quality assurance and waived the use of individual informed consent.

## Results

### Characteristics of the study population

Of the 930 OHCA patients with ROSC admitted to the study hospitals, 715 patients remained unconscious and were admitted to the study ICUs. These 715 patients constituted the cohort assessed in this study (Figure 1). Table 1 gives an overview of the demographics and clinical baseline characteristics. Coronary disease (32%), hypertension (27%), heart failure (20%), diabetes mellitus (13%) and renal impairment (3%) were the most common co-morbidities.

**Table 1:** Demographics and clinical characteristics.

	All ICUs *n *= 715	ICU A *n *= 267	ICU B *n *= 201	ICU C *n *= 247	*P *value
Age, years mean (SD)	61 (17)	59 (17)	62 (17)	63 (17)	0.006
Age >=80 *n *(%)	95 (13%)	23 (9%)	35 (17%)	37 (15%)	0.013
Male sex *n *(%)	522 (73%)	206 (77%)	153 (76%)	163 (66%)	0.009
Witnessed OHCA *n *(%)	587 (83%)	222 (83%)	166 (84%)	199 (81%)	0.627
Bystander CPR *n *(%)	458 (66%)	184 (69%)	142 (74%)	132 (55%)	<0.001
Location OHCA Home *n *(%)	331 (47%)	108 (44%)	111 (55%)	112 (45%)	0.006
EMS Response time in minutes; Mean (SD) Time to ROSC in witnessed OHCA minutes; Mean (SD)	9 (5) 26 (17)	10 (5) 30 (20)	9 (6) 26 (15)	8 (6) 21 (13)	0.007 <0.001
First rhythm shockable *n *(%)	430 (62%)	175 (66%)	126 (68%)	129 (54%)	0.003
Cardiac origin of OHCA *n *(%)	497 (73%)	202 (77%)	149 (78%)	146 (63%)	<0.001
TH *n *(%)	497 (70%)	208 (78%)	151 (76%)	138 (56%)	<0.001
EEG *n *(%) CCT *n *(%) SEP *n *(%)	166 (23%) 317 (44%) 22 (3%)	108 (40%) 128 (48%) 0 (0%)	36 (18%) 73 (36%) 20(10%)	24 (10%) 116 (47%) 2(1%)	<0.001 0.014 <0.001
Survival to discharge *n *(%)	301 (42%)	108 (41%)	89 (44%)	104 (42%)	0.747
Survival to discharge with CPC 1-2, *n *(%)	267 (37%)	94 (35%)	81 (40%)	92 (37%)	0.525

CCT= Cranial computer tomography; CPC = Cerebral performance category; EEG = Electroencephalogram; EMS = Emergency medical system; ROSC = Return of spontaneous circulation; SD = Standard deviation; SEP = Somatosensory evoked potentials; TH = Therapeutic hypothermia

The overall use of TH was 70% (Table 1). While the majority (77%) of patients with OHCA of cardiac origin were offered TH, only half (52%) of the patients with a non-cardiac origin were provided the same treatment (*P *<0.001) (Figure 1). We found the same difference in use of TH when comparing patients with an initial shockable (80%) and non-shockable rhythm (54%) (Table 2). Overall use of emergency coronary angiography and PCI in the patients with a cardiac cause (*n *= 497) was 57%.

**Table 2:** Demographics and clinical characteristics divided after initial rhythm (*n *= 715, missing data for initial rhythm 21)

	Shockable rhythm			Non-shockable rhythm		
	**TH yes (*n *= 342)**	**TH no (*n *= 87)**	***P *value**	**TH yes (*n *= 143)**	**TH no (*n *= 120)**	***P *value**

Age, years Mean (SD)	61 (15)	70 (14)	<0.001	56 (19)	62 (18)	0.005

Male sex, *n *(%)	283 (83)	58 (67)	<0.001	98 (69)	68 (57)	0.047

Witness OHCA, *n *(%)	313 (92)	(84)	0.027	101 (71)	82 (68)	0.696

Bystander CPR, *n *(%)	246 (72)	48 (55)	0.022	97 (68)	54 (45)	<0.001

Location OHCA home, *n *(%)	147 (43)	32 (37)	0.295	54 (38)	52 (43)	0.667

EMS response time, min, mean (SD)	9 (5)	9 (6)	0.519	10 (9)	9 (6)	0.360

Cardiac origin to OHCA, *n *(%)	320 (94)	76 (87)	0.120	57 (40)	34 (28)	0.050

Survival to discharge, *n *(%)	219 (64)	31 (36)	<0.001	25 (17)	11 (9)	0.051

Survival to discharge with CPC 1-2, *n *(%)	191 (56)	28 (32)	<0.001	20 (14)	9 (8)	0.094

CPC = Cerebral performance category; EMS = Emergency medical system; SD = Standard deviation; TH = Therapeutic hypothermia.

**Figure 2:**
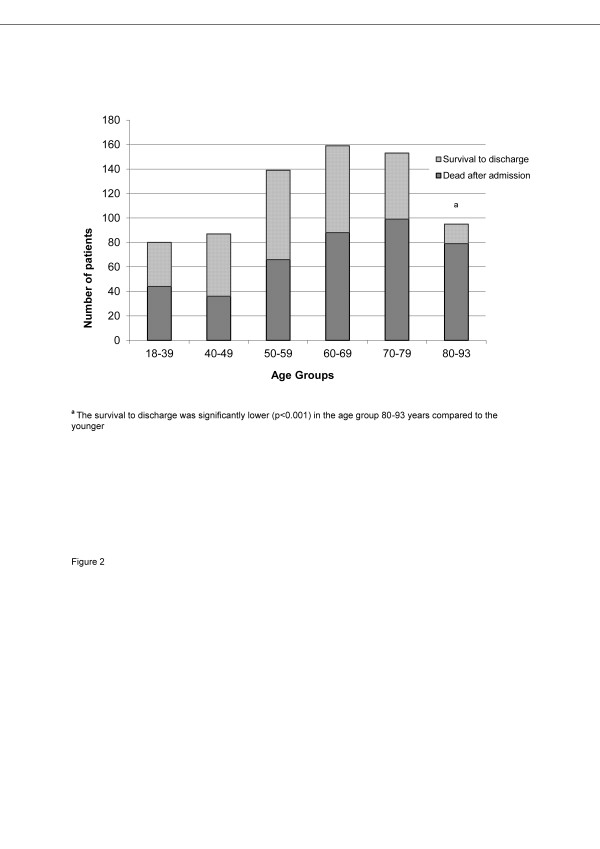
**Age distribution and survival to discharge in 715 unconscious OHCA patients admitted to the study ICUs**.

The overall survival rate to hospital discharge was 42% (Table 1). The incidence of good cerebral outcome was 90% in patients surviving to discharge. Unadjusted survival to hospital discharge and the incidence of good cerebral outcome was similar between the three sites (Table 1). Further, unadjusted survival to discharge was significantly higher in the TH treated patients (Figure 1; Table 2). The oldest patients (>80 years) had a significantly worse outcome than younger patients with only 18% of older patients surviving to hospital discharge (Figure 2). Only 33 (35%) of the 94 patients aged >80 years received TH. The corresponding number in the 31 female patients aged >80 years was five (16%). Unadjusted survival in patients aged >80 years who received TH was not significantly higher than those that did not receive TH (21% *vs*. 15%, respectively, *P *= 0.426).

Overall, survivors had a significantly longer median length of ventilator treatment than non-survivors: 65 h (41-133 h) *versus *52 h (18-111 h) (*P *= 0.001). Similarly, survivors also had a significantly longer median ICU stay (6 days *versus *3 days, *P *<0.001).

### Predictors for use of TH and survival

In a multivariable logistic regression model, we found witnessed arrest, an initial shockable rhythm, cardiac origin and bystander CPR to be positive predictors for the use of TH (Table 3a). On the other hand, increasing age and female gender predicted a lower utilisation of TH (Table 3a). After dividing the study population in two groups according to initial rhythm (Table 3b and 3c), we found that increasing age and female gender remained negative predictors for TH use in the subgroup of patients with shockable rhythm (Table 3b). In the subgroup of patients with non-shockable rhythms, however, female gender did not remain a significant predictor for TH use, while study ICU became a significant predictor (Table 3c).

**Table 3a:** Predictors for the use of therapeutic hypothermia in all patients (*n *= 715; missing data 68).

	OR	95% CI	*P *value
Age (one additional year)	0.96	0.94-0.97	<0.001
Witnessed arrest	2.08	1.26-3.40	0.004
Female gender	0.65	0.43-0.97	0.038
Bystander CPR	2.30	1.57-3.39	0.001
Shockable rhythm	1.92	1.20-3.09	0.007
Cardiac origin	2.92	1.70-5.02	<0.001

CPR = Cardiopulmonary resuscitation; multivariable logistic regression model with adjusted odds ratio (OR)

**Table 3b:** Predictors for the use of therapeutic hypothermia in patients with shockable rhythm (*n *= 421; missing data 9)

	OR	95% CI	*P *value
Age (one additional year)	0.95	0.93-0.97	<0.001
Witnessed arrest	2.36	1.11-4.99	0.025
Female gender	0.48	0.28-0.86	0.013
Bystander CPR	1.99	1.18-3.35	<0.001

CPR = Cardiopulmonary resuscitation; multivariable logistic regression model with adjusted odds ratio (OR)

**Table 3c:** Predictors for the use of therapeutic hypothermia in patients with a non-shockable rhythm (*n *= 235; missing data 29)

	OR	95% CI	*P *value
Age(one additional year)	0.97	0.95-0.99	0.001
Bystander CPR	2.36	1.33-4.2	0.003
Cardiac origin	2.89	1.48-5.66	0.002
ICU - Reference A ICU B	0.8	0.36-1.77	0.79
ICU C	0.36	0.19-0.68	0.002

CPR = Cardiopulmonary resuscitation; multivariable logistic regression model with adjusted odds ratio (OR)

In terms of survival, cardiac origin and an initial shockable rhythm were positive predictors of survival, while increasing age, female gender and diabetes mellitus were negative predictors of survival (Table 4). When analysing data from patients with witnessed OHCA only (*n *= 587; 82% of all patients), we could also include time to ROSC as a variable in the logistic regression model. Doing this, study site as a significant positive predictor for survival (Table 4) to discharge disappeared while time to ROSC became a significant factor (Table 5). The predictive power of the other factors, including TH, remained the same (Table 5).

**Table 4:** Predictors of survival to discharge in all patients (*n *= 715; missing data 96)

	OR	95% CI	*P *value
Age (one additional year)	0.96	0.94-0.97	<0.001
Female gender	0.57	0.36-0.92	0.022
Cardiac origin	2.64	1.36-5.10	0.001
Diabetes mellitus	0.35	0.19-0.65	0.001
Shockable rhythm	6.50	3.77-11.38	<0.001
TH	1.91	1.18-3.06	0.007
ICU - Reference A ICU B	1.08	0.68-1.72	0.732
ICU C	2.35	1.46-3.75	<0.001

Multivariable logistic regression model with adjusted odds ratio (OR); TH = Therapeutic hypothermia

**Table 5:** Predictors of survival to discharge in patients with witnessed OHCA (*n *= 587; missing data 92)

	OR	95% CI	*P *value
Age (one additional year)	0.95	0.93-0.96	<0.001
Female gender	0.47	0.27-0.80	0.007
Cardiac origin	3.11	1.43-6.77	0.004
Diabetes mellitus	0.47	0.24-0.92	0.029
Shockable rhythm	5.77	3.04-10.94	<0.001
Time to ROSC (one additional minute)	0.95	0.93-0.96	<0.001
TH	1.87	1.05-3.31	0.033

Multivariable logistic regression model with adjusted odds ratio (OR); ROSC = Return of spontaneous circulation; TH = Therapeutic hypothermia

When testing the significance of clinical relevant interactions between variables used in the logistic regression analyses, we found a significant interaction between shockable rhythm and gender. For males the OR for survival for shockable rhythm was 9.01 (95% CI 4.80 to 16.91), while for females the same OR was 2.75 (95% CI 1.14 to 6.62). When including this interaction term in the logistic regression model there is no further significant effect of sex and the effect of the other variables in Table 4 remained unchanged. We did not find any such interactions for study site. Further, there was not a significant interaction between TH and initial rhythm for survival.

Because the application of TH was based on the treating physician's decision and not random, we also included a propensity score for use of TH as a covariate in the multivariable analysis of possible predictors of survival to discharge. However, this did not change the adjusted OR for the effect of TH compared to the analysis above (Table 4 and 5) and is therefore not further reported.

When analysing factors predicting survival in patients with shockable and non-shockable rhythm separately, the overall results for patients with shockable rhythm (*n *= 430) did not change compared to the total cohort. In the smaller subgroup of patients with a non-shockable first rhythm (*n *= 264), however, use of TH (OR 1.46; 95% CI 0.56 to 3.78) did not remain a significant predictor for survival any longer. Age (OR 0.97; 95% CI 0.95 to 0.99) and cardiac origin (OR 2.95; 95% CI 0.1.27 to 6.99) were the only independent predictors for survival to discharge in this subgroup.

## Discussion

We studied the use of TH and related outcomes in unconscious patients who suffered an OHCA from either cardiac or non-cardiac causes and were admitted to the ICU. TH was used in 70% of the patients. However, patients with an initial non-shockable rhythm or a non-cardiac cause of the OHCA were significantly less likely to receive TH. The overall survival to discharge was 42%, with 90% of patients surviving to discharge having a good neurologic outcome, defined as a CPC of 1-2 [17]. Known positive prognostic factors, such as a witnessed arrest, bystander CPR, initial shockable rhythm and cardiac cause of OHCA were all positive predictors of TH use as well as survival.

After correcting for the other prognostic factors, the use of TH was an independent predictor of improved survival in the whole study population. However, when performing a subgroup analysis of patients with non-shockable rhythms, use of TH did not remain a significant predictor of survival. One reason for this can be lower power of the analysis due to the inevitably lower number of patients in such subgroup analysis. It is also important to emphasise that the expected survival rate and thereby the potential impact of TH is much lower for patients with a non-shockable rhythm, as seen both in our study and other reports [22-25]. Some authors have found no benefits of TH in patients with non-shockable rhythm [26,27] while others have concluded that a positive impact on survival is likely [23,25,28,29]. The use of TH in such patients was recommended by ILCOR both in 2005 and 2010 [30,31]. In Norway, the use of TH in patients with an initial non-shockable rhythm has been recommended since 2004 [8], but only 50% of such patients were actually cooled in our study. In a recent study covering all Finnish ICUs, the authors detected large and unexplained differences in local cooling practice [24]. They also found use of TH in one-third of patients with an initial non-shockable rhythm, despite the national recommendation not to cool such patients [24]. We think this underlines the present uncertainty of local cooling practices and its effect on outcome. We need more and larger studies to fully understand the treatment potential of TH in various subgroups of post-resuscitation patients [23].

We found that increasing age predicted less use of TH, but also a lower survival. In the recent FINNRESUSCI study, advanced age was a common reason for withholding TH in unconscious OHCA patients [24]. The evidence showing benefits of TH in older patients is sparse. In a single-centre study of TH in patients fulfilling the criteria for inclusion in the original HACA trial [1], older age (≥80 years) was associated with a lower survival [32]. Still, 50% of the older patients undergoing TH survived with good cerebral outcome. Previous studies have shown that emergency and intensive care physicians are reluctant to use ICU resources in older patients, and it has been discussed whether this represent age discrimination or sensible resource allocation [33,34]. It may be argued that TH use in the present study was not associated with a higher survival rate in the oldest age group (≥80 years). However, the total number of older patients treated with TH was low, and there are still healthy older patients that might benefit from TH. Therefore, a more liberal use of TH may be indicated [34].

Surprisingly, we found female gender to be an independent predictor both for decreased TH use and survival in patients with a shockable rhythm. Previous studies have found lower utilisation of invasive procedures, such as percutaneous coronary intervention, in female patients [35]. Different age distributions and co-morbidity related to gender may partly explain the differences in care provided and outcome in women *versus *men [36-38]. Recent studies in the ICU setting have provided conflicting results regarding interaction between gender, medical interventions and survival [39,40]. We are not aware of any previous studies looking at this aspect in post cardiac arrest patients. Since we corrected for age, prehospital cardiac arrest factors and co-morbidity in our logistic regression model, it is concerning that female gender remained a negative predictor of survival in patients with a shockable rhythm. In contrast, recent prehospital studies reported female gender to either be a positive predictor of survival or be without predictive power [41,42]. Gender has been highlighted as an important factor to address when trying to improve overall cardiac care [38]. Our results may indicate underutilisation of TH in female patients. Our results should definitely lead to more research on the impact of age and gender on the use of TH and the associated outcomes.

We found study site to be an independent predictor for the use of TH in the subgroup of patients with non-shockable rhythm. This finding is reflected in the overall lower use of TH in this ICU. Study site was also an independent predictor of survival in the logistic regression model including the whole cohort. A recent German study [43] found significant differences in survival in OHCA patients depending on what kind of hospital they were brought to. All our hospitals offered emergency coronary angiography and were by definition cardiac arrest centres [44]. Differences in prehospital factors [45] are therefore a more likely explanation for the difference in survival [45]. Compared to the other two ICUs, patients from the ICU with the improved odds of survival had significantly shorter EMS response times, as well as shorter times to ROSC. Time to ROSC is a very important predictor of survival but only available in patients with witnessed OHCA [46]. We therefore analysed patients with witnessed OHCA (82% of total cohort) separately. Doing this, we found that time to ROSC but not study site remained a significant predictor of survival. We think this finding strongly indicates that differences in prehospital factors affected hospital mortality in our study. Comatose OHCA patients most likely entered the ICU with a different baseline probability of survival due to differences in resuscitation times [45-48].

Since the use of TH was at the physician discretion in individual patients we tried to adjust for factors predicting the use of TH in the individual patient by introducing a propensity score as a covariate in the logistic regression model on survival [20,21].However, this did not change the estimated effect of TH. Nevertheless, the observational study design only allowed us to show statistical associations and not prove causative relationships. Only prospective, randomised controlled trials can prove the efficacy of TH in subgroups of patients with OHCA admitted to the ICU. Still, well-designed observational studies may provide insight into everyday use of TH and help improve clinical practice [49].

### Limitations

There are several limitations to our retrospective and registry based study. Despite an agreement on definitions for the different data points, data verification may have varied from site to site. This may have changed the grouping of patients and affected the logistic regression model. Although we adjusted for other known factors when modelling the relation between use of TH and survival, the validity of the resulting models may have been weakened by unknown or unobserved factors not included. Further, despite the similarities in organisation of the ICUs studied, there may have been differences in the medical management and hospital policy not accounted for. It is known that withdrawal of life-sustaining therapy is a major factor of death in post cardiac arrest care and that early withdrawal of intensive care may be a problem [48,50]. The overall low use of objective prognostic tools in our patients was in accordance with common practice in Norwegian ICUs at the time of the study [4]. Still, we cannot fully rule out differences in clinical management that may have affected patient outcome in the study ICUs. We defined TH as reaching the targeted temperature for 12-24 h. TH may have been attempted unsuccessfully in more patients. The fact that we only followed patients to hospital discharge may also be considered a limitation of the present study.

Finally, we studied patients from three different cardiac arrest centres [44], but only from one country. The generalisability of our results to other countries and hospital systems may be questioned. Still, compared to other registry-based studies [46,51], a major strength of our study is that we included consecutively admitted comatose patients over a defined time period.

## Conclusion

TH was used in the majority of unconscious OHCA patients admitted to the ICU, but actual use varied significantly between subgroups. Increasing age predicted both a decreased utilisation of TH as well as a lower survival. Further, in patients with a shockable rhythm female gender also predicted a lower use of TH and poorer survival. Our results indicate an underutilisation of TH in some subgroups. Hence, more research on factors affecting TH use and the associated outcomes in subgroups of post-resuscitation patients is needed.

## Key messages

- Older age predicted a lower use of therapeutic hypothermia as well as a lower survival in unconscious OHCA patients admitted to the ICU.

- In patients with an initial shockable rhythm, female gender predicted a lower utilisation of therapeutic hypothermia and a lower survival to hospital discharge.

- The role of therapeutic hypothermia in patients with an initial non-shockable rhythm is still not fully elucidated.

## Abbreviations

CI: Confidence interval; CPR: Cardio pulmonary resuscitation; ICU: Intensive care unit; OHCA: Out-of-hospital cardiac arrest; OR: Odds ratio; PCI: Percutaneous coronary intervention; ROSC: Return-of-spontaneous circulation; TH: Therapeutic hypothermia

## Competing interests

The authors declare that they have no competing interests.

## Authors' contributions

TWL contributed to the design of the study, took part in the data acquisition, built the database and performed the statistical analysis, and drafted the manuscript. JL and KS made substantial contribution to the design of the study took part in data acquisition and helped draft the manuscript. JTK contributed to the study design, provided statistical support and helped draft the manuscript. AIL, JKH and TD contributed to the design of the study, took part in data acquisition and helped draft the manuscript. ES conceived of the study, participated in its design, coordination and statistical analysis, and helped draft the manuscript. All authors read and approved the final manuscript for publication.
